# Anal HIV DNA is associated with high-risk HPV genotypes in anal cytology specimens obtained for anal neoplasia screening

**DOI:** 10.1186/1750-9378-7-S1-P4

**Published:** 2012-04-19

**Authors:** Bruce Shiramizu, Chin-Yuan Liang, Xumei Zhu, Melissa Agsalada, Ian Nagata, Kellie Kitamura, Jeffrey Killeen, J Michael Berry, Marc Goodman

**Affiliations:** 1 University of Hawaii, Hawaii Center of AIDS, Honolulu, HI, USA; 2 University of Hawaii Cancer Center, Honolulu, HI, USA; 3 University of Hawaii, Hawaii Department of Pathology, Honolulu, HI, USA; 4 University of California, San Francisco, CA, USA

## Purpose

High-grade anal neoplasia (AN) is associated with high-risk human papillomavirus (HPV) genotypes and is the precursor to anal cancer. Individuals infected with human immunodeficiency virus type 1 (HIV) continue to be at increased risk for AN even while on effective antiretroviral therapy (ART) with undetectable HIV RNA levels. The study was undertaken to assess HIV DNA from anal cytology specimens to determine if HIV DNA copy number was a factor for presence of high risk HPV genotypes.

## Material and methods

Anal cytology specimens were obtained as part of an AN study according to guidelines approved by the local institutional review board. Anal HPV genotype, HIV DNA copy numbers, and cytology were obtained for each specimen. High-risk HPV genotypes included 16, 18, 31, 33, 35, 39, 45, 51, 52, 56, 58, 59, and 68. Analysis was performed by logistic regression model with high HPV risk as the response and HIV DNA copy numbers, anal Pap cytology results, and nadir CD4 cell counts as predictors. Lemeshow goodness-of-fit test was then performed to check model fit. Similar procedure was performed in sub-cohort of undetectable HIV RNA patients.

## Results

**Figure 1:**
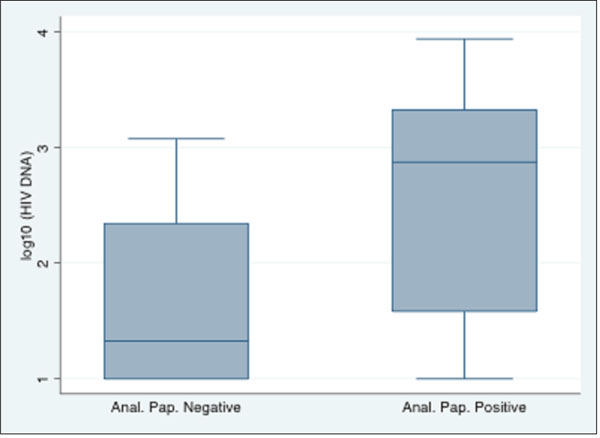
Differences in HIV DNA distributions between normal and atypical anal Pap groups (p=0.011).

46 specimens were available from 38 males and 8 females. 52.2% of the specimens were negative for any HPV with 52.6% of males being HPV-positive compared to 25% of females. Of 46 specimens, 42 patients had undetectable HIV RNA level. The odds of having high-risk HPV genotypes among subjects with (x+100) copies of HIV DNA was 1.161 times the odds among subjects with x copies of HIV DNA (p=0.013). Inclusion of nadir CD4 count in the logistic regression model did not predict HPV risk. Distributions of HIV DNA were statistically different between normal and abnormal anal cytologies (p=0.009); median and interquartile HIV DNA copy numbers for abnormal and normal cytologies 792 (38-2100) and 27.5 (10-225), respectively, Figure 1. Anal cytology results were also associated with HPV risk (p=0.034). Patients with undetectable HIV RNA (n=42) had similar findings.

## Conclusions

Individuals with higher HIV DNA copies in anal specimens were more likely to have high-risk HPV genotypes independent of nadir CD4 cell count. Abnormal anal cytologies were also associated with high-risk HPV. The association of HIV DNA copy number in anal specimens needs validation in future studies to determine the role in the pathogenesis of AN and high risk HPV. Grant support: RR011091, RR026136, CA121947, CA143727, CA096254.

